# Ending TB: the world’s oldest pandemic

**DOI:** 10.1002/jia2.25698

**Published:** 2021-03-23

**Authors:** Peter S Kim, Soumya Swaminathan

**Affiliations:** ^1^ Division of AIDS National Institute of Allergy and Infectious Diseases National Institutes of Health Bethesda MD USA; ^2^ World Health Organization Geneva Switzerland

**Keywords:** tuberculosis, mycobacterium tuberculosis, TB, pandemic, epidemic

Few diseases have impacted human history as much as tuberculosis (TB). *Mycobacterium tuberculosis* (MTB) has caused disease in humans for more than 4000 years [1]. Since its scientific discovery in 1882, over 1 billion people have died from TB – a death toll greater than that from malaria, smallpox, HIV/AIDS, cholera, plague and influenza combined. Today, over 2 billion people are latently infected, 10 million people have TB, and TB is the leading infectious cause of death worldwide, besides COVID‐19.

Though advances in TB research have historically been very slow, recent efforts undergirded by increased research funding and political determination have realized significant gains. There are currently almost 20 new drugs in the clinical development pipeline, multiple new vaccine candidates, an array of new diagnostics and multiple clinical trials characterizing the efficacy and effectiveness of these new tools.

For chemoprophylaxis and treatment, several new regimens have proven successful in simplifying complex therapeutic requirements. The PREVENT TB Study showed that three months of once‐weekly therapy with rifapentine plus isoniazid was as effective as nine months of isoniazid in preventing TB [2]. The BRIEF TB study demonstrated that a one‐month regimen of daily rifapentine plus isoniazid was non‐inferior to nine months of isoniazid for preventing TB in adults and adolescents with HIV [3]. The NIX TB trial showed that a six‐month combination regimen of three oral drugs – bedaquiline, pretomanid and linezolid – was safe and effective for treating highly drug‐resistant TB [4]. Most recently, a collaborative trial between the U.S. TB Trials Consortium and the AIDS Clinical Trials Group (Study 31/A5349) showed that a four‐month regimen of high‐dose rifapentine, isoniazid, pyrazinamide and moxifloxacin was non‐inferior to the current standard six‐month regimen – a reduction in treatment duration that may have significant implications for global treatment efforts [5].

Similarly, advances in diagnostics such as the Xpert^®^ MTB/RIF Ultra assay have enabled more timely diagnosis of TB and associated drug resistance. In a recent study, the Xpert^®^ MTB/XDR assay showed sensitivity and specificity comparable to phenotypic testing for isoniazid, fluoroquinolones, ethionamide and second‐line injectable drugs in under 90 minutes [6]. Additionally, efforts are underway to improve antigen detection technologies such as the urine lipoarabinomannan lateral flow assay which enables rapid diagnosis of TB in people living with HIV. Efforts to further delineate mycobacterial genetic mutations associated with drug resistance and host‐derived immune signatures representative of the spectrum of TB infection and disease are increasingly successful, and portend holistic diagnostic strategies that enable more accurate diagnosis and improved care.

With regard to TB vaccines, recent studies highlight the potential of new and old vaccine candidates alike. A phase 2 trial evaluating the efficacy of H4:IC31, a candidate subunit vaccine, and the Bacillus Calmette–Guérin (BCG) vaccine to prevent MTB infection among adolescents found that BCG may be effective in preventing persistent MTB infection [7]. A phase 2b clinical trial of the M72/AS01E vaccine demonstrated 49.7% efficacy in preventing the development of active pulmonary TB for three years among those with latent MTB infection [8]. Most recently, a preclinical study evaluating intravenous administration of BCG in rhesus macaques found that the strategy prevented the acquisition of infection after aerosol challenge with MTB [9].

These studies represent the most significant advances in TB vaccinology since the advent of the BCG vaccine, and indicate that a successful vaccine may be achievable in the near future. Several additional promising vaccine candidates are in clinical development, including VPM1002 [10, 11], MTBVAC [12] and ID93:GLA‐SE [13, 14]. Research efforts to identify correlates of protection, develop improved adjuvants and enhance understanding of protective immune mechanisms are ongoing and will provide the basis for the discovery and development of future vaccine candidates.

Despite these advances, much remains to be done. In 2019 alone, TB caused 1.5 million deaths, including 251,000 deaths among persons with HIV. Millions of cases went undiagnosed, and only one out of three people with drug‐resistant TB received appropriate treatment. The prevalence of drug‐resistant TB continues to grow. The tools and strategies needed to bring an end to the TB pandemic, whereas on the horizon, are not yet available. Effective vaccines, true point‐of‐care diagnostics, simpler treatment regimens and strategies to improve case finding and treatment retention are attainable. However, success requires redoubling of investment in research and research infrastructure backed by political support to ensure that achievements to date are not lost.

International research collaborations have the potential to enable economies of scale, leverage expertise from multiple researchers and institutions, maximize efficient use of scarce resources, foster development of global research capacity and speed translation of research discoveries. Most of the recent scientific breakthroughs in TB have resulted from global collaborations. At the same time, there is a great need to potentiate international research by removing legal and bureaucratic hurdles that prohibit the free flow of research ideas, data and samples, thereby delaying or obstructing critical collaborations. Efforts that encourage cutting‐edge collaborative science, enable pathways for trans‐national data and sample sharing, and foster development of research capacity within LMICs, especially engaging early‐career investigators, are urgently needed. GISAID and GenBank are data‐sharing platforms through which influenza and SARS‐CoV‐2 sequence data and related clinical and epidemiological data can be shared openly by the research community [15, 16]. The TB Portals programme is a global repository of TB case data with a suite of analytic tools available to the public [17]. Platforms such as the GISAID Initiative, GenBank and TB Portals should be leveraged to create a global database of mycobacterial genomic data paired with phenotypic and clinical data to rapidly advance mycobacterial research. Networks such as RePORT International [18], which foster global research collaboration while building local research capacity, should be championed by governments to remove constraints on trans‐national collaboration.

The COVID‐19 pandemic has shown that research and development resulting in improved clinical care can be dramatically accelerated. We are beneficiaries of rapid information sharing and enhanced global collaboration. We have seen that new vaccines, treatments and diagnostics can be rapidly developed when scientific pursuits are fully supported financially and politically and fostered through well‐financed public–private partnerships. Exemplified by the mRNA vaccines against COVID‐19, advances have not only been a re‐engineering of old technology, but pioneering breakthroughs. As a result, the world has a growing armamentarium of safe and effective medical countermeasures against COVID‐19. All this for a pandemic that is a little over one year old. These discoveries and the methods used to achieve them have paved the way for other infectious diseases, including TB. It is time that we apply these same methods and intensity to the oldest pandemic of them all.

## COMPETING INTERESTS

The authors have declared no conflict of interest.

## AUTHORS’ CONTRIBUTIONS

Dr. Peter Kim and Dr. Soumya Swaminathan devised the manuscript together.

## AUTHOR INFORMATION

Dr. Peter Kim is the Director of the Therapeutic Research Program in the Division of AIDS, National Institute of Allergy and Infectious Disease, U.S. National Institutes of Health. Dr. Soumya Swaminathan is the Chief Scientist of the World Health Organization.
